# Enhanced superconductivity in atomically thin TaS_2_

**DOI:** 10.1038/ncomms11043

**Published:** 2016-03-17

**Authors:** Efrén Navarro-Moratalla, Joshua O. Island, Samuel Mañas-Valero, Elena Pinilla-Cienfuegos, Andres Castellanos-Gomez, Jorge Quereda, Gabino Rubio-Bollinger, Luca Chirolli, Jose Angel Silva-Guillén, Nicolás Agraït, Gary A. Steele, Francisco Guinea, Herre S. J. van der Zant, Eugenio Coronado

**Affiliations:** 1 Universidad de Valencia (ICMol), Catedrático José Beltrán Martínez n° 2, Paterna 46980, Spain; 2 Kavli Institute of Nanoscience, Delft University of Technology, Lorentzweg 1, Delft 2628 CJ, The Netherlands; 3 Departamento de Física de la Materia Condensada, Universidad Autónoma de Madrid, Campus de Cantoblanco, Madrid 28049, Spain; 4 Condensed Matter Physics Center (IFIMAC), Universidad Autónoma de Madrid, Madrid 28049, Spain; 5 Instituto Madrileño de Estudios Avanzados en Nanociencia (IMDEA- Nanociencia), Calle Farady 9, Cantoblanco, Madrid 28049, Spain

## Abstract

The ability to exfoliate layered materials down to the single layer limit has presented the opportunity to understand how a gradual reduction in dimensionality affects the properties of bulk materials. Here we use this top–down approach to address the problem of superconductivity in the two-dimensional limit. The transport properties of electronic devices based on 2H tantalum disulfide flakes of different thicknesses are presented. We observe that superconductivity persists down to the thinnest layer investigated (3.5 nm), and interestingly, we find a pronounced enhancement in the critical temperature from 0.5 to 2.2 K as the layers are thinned down. In addition, we propose a tight-binding model, which allows us to attribute this phenomenon to an enhancement of the effective electron–phonon coupling constant. This work provides evidence that reducing the dimensionality can strengthen superconductivity as opposed to the weakening effect that has been reported in other 2D materials so far.

## Abstract (web summary)

As a material's thickness decreases towards the atomic-scale, dimensional confinement may promote behaviour not found in the bulk, with potential technological applications. Here, the authors study superconductivity in TaS_2_ as it is mechanically exfoliated towards the two-dimensional limit.

## Graphical abstract

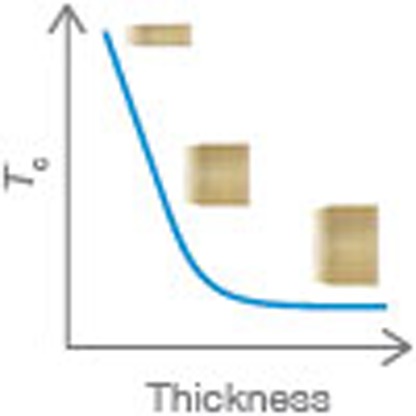

The behaviour of superconductors in the two-dimensional (2D) limit is a long-standing problem in physics that has been the focus of extensive research in the field^1^,^2^,^3^,^4^,^5^,^6^. The bottom–up approach has provided signs of the existence of superconductivity at the 2D limit in experiments performed on *in situ*-grown, ultrathin lead films fabricated by evaporation^7^,^8^. However, for films grown in this way, it is difficult to avoid the strong influence from the substrate lattice, yielding typically highly disordered films. A different approach takes advantage of the ability of certain van der Waals materials to be separated into individual layers, which may later be isolated as defect-free 2D crystals on a substrate of choice^9^. This top–down approach permits overcoming the lattice and chemical restrictions imposed by the substrate in the bottom–up strategy in such a way that the coupling may be minimized by an appropriate choice of surface^10^,^11^,^12^.

Although graphene is not an intrinsic superconductor, recent studies have brought forward the possibility of inducing superconductivity in this 2D material by garnishing its surface with the right species of dopant atoms or, alternatively, by using ionic liquid gating^13^,^14^. However, reported experiments have failed to show direct evidence of superconducting behaviour in exfoliated graphene, leaving out the archetypal material from studies of 2D superconductivity^15^.

An even more attractive family of 2D materials is provided by the transition metal dichalcogenides (TMDCs) since some of its members exhibit superconductivity in the bulk state^16^,^17^. Just as in graphene, TMDCs present a strong in-plane covalency and weak interlayer van der Waals interactions, which allow exfoliation of the bulk^18^. This has given rise to a very rich chemistry of hybrid multifunctional materials based on the restacking of TMDC nano-layer sols with functional counterparts^19^,^20^. In addition, the all-dry exfoliation methodologies have allowed for the deposition of TMDC flakes on a variety of surfaces^21^,^22^. These micromechanical exfoliation techniques allow access to nearly defect-free, large surface area flakes of virtually any TMDC, opening the door to the study of how a dimensionality reduction affects the properties of these materials^23^,^24^,^25^,^26^. Surprisingly, despite the works reported in the literature searching for intrinsic superconductivity in atomically thin 2D crystals^27^,^28^,^29^, for a long time the sole examples came from FeSe thin films grown *in situ* on a substrate^30^,^31^,^32^,^33^. Only very recently, several studies of niobium diselenide (NbSe_2_) flakes have yielded the first clear evidence of the existence of superconductivity in freshly cleaved specimens of less than three layers in thickness^34^,^35^,^36^,^37^.

Tantalum disulphide (TaS_2_) is another member of the TMDC family. In its bulk state, TaS_2_ is composed of robust covalently bonded S–Ta–S planes that stack upon each other. A variety of polytypic phases originate from the distinct in-plane Ta coordination spheres described by the S^2−^ ligands and by the stacking periodicity of the individual planes. For instance, the 2H and 1T polytypes present unit cells with two trigonal bipyramidal and one octahedral Ta-coordinated layers, respectively. Although extensively explored in the 1960s^38^, 1T and 2H polytypes are once again attracting major attention as they constitute ideal case studies for the investigation of competing orders, namely, superconductivity, charge density waves (CDW)^39^,^40^ and hidden phases^41^. In this scenario, the study of decoupled or isolated TaS_2_ layers may provide new insights into these exotic phenomena^42^. Transport measurements of few-layer TaS_2_ flakes have been reported in flakes as thin as 2 nm, but superconductivity in TaS_2_ layers thinner than 8 nm has not been observed, probably due to the environmental degradation of the samples^43^.

Here, we explore 2D superconductivity in few-molecular-layer tantalum disulphide flakes of different thicknesses, which have been mechanically exfoliated onto Si/SiO_2_ substrates. Interestingly, we observe that superconductivity persists down to the thinnest layer investigated (3.5 nm, approximately 5 covalent planes), with a pronounced increase in the critical temperature (*T*_c_) from 0.5 K (bulk crystal) to ∼2.2 K when the thickness of the layer is decreased. In search of the origin of these observations, we perform density functional theory (DFT) calculations and construct a simple tight binding model to study the change in the electronic band structure and density of states (DOSs) at the Fermi level as a function of reduced thickness. We ascribe the enhancement to an increase in the effective coupling constant (*λ*_eff_) for reduced thicknesses, which ultimately determines *T*_c_.

## Results

### Fabrication of transport devices

Although the exfoliation of other TMDC members has been extensively studied, little has been reported on the controlled isolation of atomically thin 2H-TaS_2_ flakes. This layered material appears to be difficult to exfoliate and is also particularly susceptible to oxidation in atmospheric conditions^44^, hindering the manipulation of very thin flakes in open moist air. Although complex encapsulation techniques help preserving samples from oxidation^36^, we find that a rapid integration of freshly exfoliated flakes into final devices and their immediate transfer to vacuum conditions for measurement also permits retaining the pristine properties of most TaS_2_ samples (vide infra).

**Figure 1:**
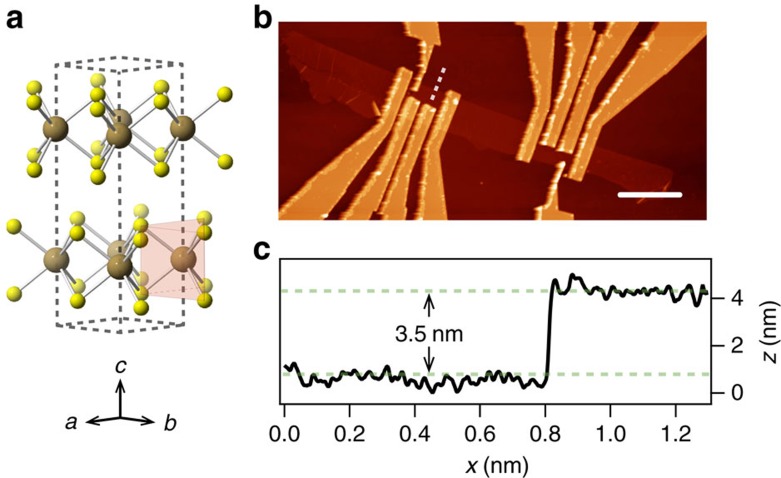
Atomically thin TaS_2_ devices. (**a**) Ball and stick model of the crystal structure of the 2H polytype of TaS_2_. The dashed prism encloses the content of a single unit cell and the metal coordination geometry is highlighted by the red polyhedron. (**b**) Atomic force microscopy image of two devices fabricated on a 3.5-nm 2H-TaS_2_ flake. The scale bar is 4 μm in length. The full colour scale of the topograph corresponds to a height of 100 nm. (**c**) Line profile of the flake taken at the location of the white dotted line in **b**.

The experimental process begins with the chemical vapour transport growth of bulk TaS_2_ crystals (vide infra in Methods), which are subsequently exfoliated onto Si/SiO_2_ substrates. To ensure a high-quality material, optical, Raman and atomic force microscopy characterization were performed on exfoliated flakes of varying thicknesses (see Supplementary Figs 1–5 and Supplementary Note 1 and 2 for details). As already established for graphene and other TMDCs, inspection of the substrate surface by optical microscopy permits identifying the presence of nanometre thin TaS_2_ flakes. In an attempt to access flakes with a reduced number of atomic layers, we developed a modification of the micromechanical exfoliation method and optimized it for the controlled isolation of few-layer 2H-TaS_2_ flakes^45^,^46^. The method relies on precisely controlling a uniaxial pressure applied directly with a single crystal over the accepting substrate and in combination with a shearing cleavage movement. This allows for the cleavage of very thin flakes, down to 1.2 nm thick (see Supplementary Fig. 1 for details), corresponding to a single 2H-TaS_2_ unit-cell (see Fig. 1a) formed by two individual layers. Unfortunately, all attempts to contact these flakes and measure transport properties were unsuccessful, likely due to their instability in ambient conditions.

To avoid the oxidation of few layer flakes, freshly exfoliated samples designated for device fabrication and transport measurements are immediately covered with an methyl methacrylate/poly (methyl methacrylate) double layer resist in preparation for subsequent device nanofabrication steps. Figure 1b shows an example of a fabricated device incorporating the thinnest flake measured with a thickness of 3.5 nm (corresponding to approximately 5 layers) and lateral dimensions of the order of a few micrometres as imaged by atomic force microscopy. The chromium/gold (Cr/Au, 5 nm/70 nm) electrodes were evaporated onto selected flakes by employing standard e-beam lithography techniques (see Methods for details). All transport measurements were made using a four terminal current bias configuration in a temperature range of 20 mK to 4 K in a dilution fridge.

### Transport properties and superconductivity

**Figure 2:**
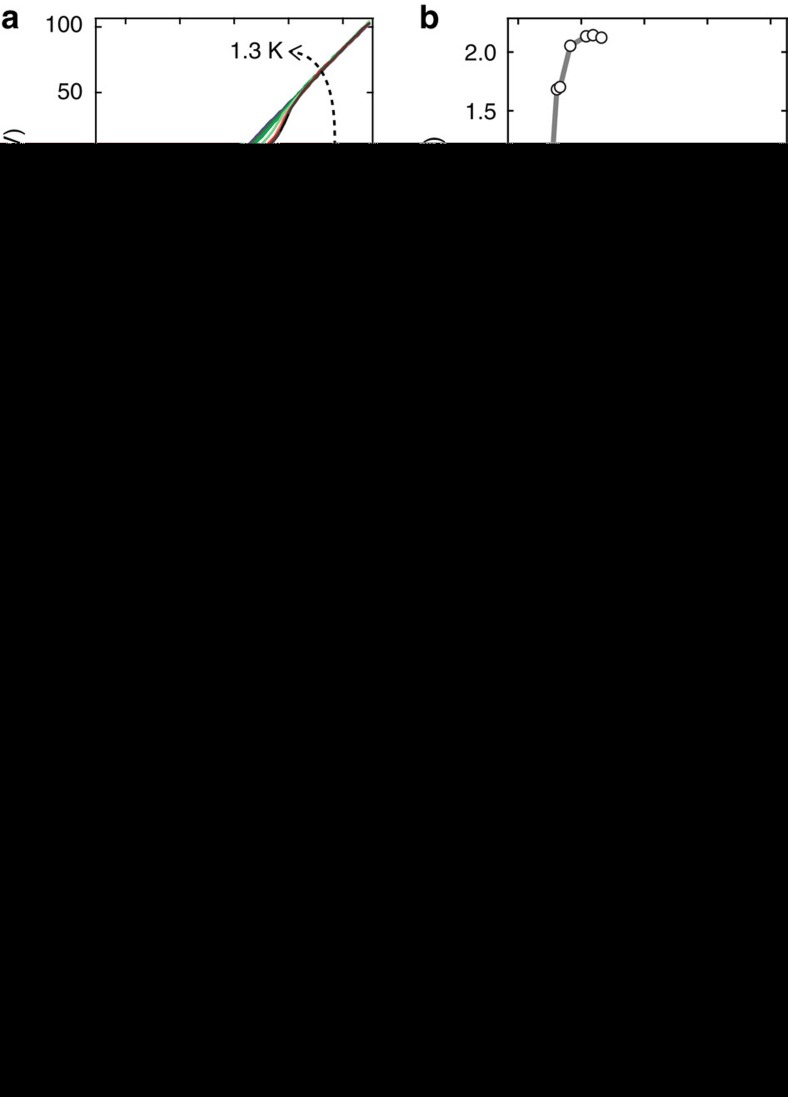
Superconductivity in atomically thin crystals. Temperature dependence of three selected devices spanning the range of thicknesses studied. (**a**) Current–voltage (*I*–*V*) characteristics as a function of temperature for a bulk-like 14.9 nm device. (**b**) Resistance (zero bias numerical derivative) versus temperature for the 14.9 nm device. (**c**) *I*–*V* characteristics as a function of temperature for a 5.8-nm device. (**d**) Resistance versus temperature for the 5.8-nm device. (**e**) *I*–*V* characteristics as a function of temperature for a 4.2-nm device. (**f**) Resistance versus temperature for the 4.2-nm device.

We present measurements on 12 flakes of varying thicknesses in the ≈3–30 nm range, integrated in the described four-terminal devices, with the aim of studying the effect of dimensionality reduction on the superconducting properties of TaS_2_. All devices show a superconducting transition observed by four terminal current bias measurements as a function of temperature. Figure 2 shows the current–voltage (*I*–*V*) and resistance–temperature (*R*–*T*) characteristics for three representative devices having thicknesses of 14.9 nm (Fig. 2a,b), 5.8 nm (Fig. 2c,d) and 4.2 nm (Fig. 2e,f). The transport data for the thinnest 3.5 nm flake can be found in the Supplementary Fig. 6. The zero bias, numerical derivatives (d*V*/d*I*) as a function of temperature show a clear superconducting transition for each device (Fig. 2b,d,f). From these (interpolated) curves, we estimate *T*_c_, taken at 50% of the normal-state resistance. For the 14.9 nm flake, and despite the fact that the sample does not attain a zero resistive state, one may still appreciate that there is a phase transition centred at 540±230 mK. This is in rough agreement with previously reported *T*_c_ values of 600 mK for bulk 2H-TaS_2_ material^47^. Interestingly, and in contrast with studies on other 2D superconductors, the *T*_c_ values show a marked increase for the thinner flakes of 5.8 nm (1.45±0.13 K) and 4.2 nm (1.79±0.20 K). This peculiar result is discussed in detail below. In addition, critical current densities increase by orders of magnitude as the devices become thinner (14.9 nm, *J*_c_≈700 A cm^−2^, 5.8 nm, *J*_c_≈7 × 10^4^ A cm^−2^ and 4.2 nm, *J*_c_≈5 × 10^5^ A cm^−2^). In thin film superconductors with high critical current densities, as those measured in our thinnest flakes, Joule self-heating starts to play a role^48^. This explains the pronounced asymmetry in the *I*–*V* characteristics for thinner flakes (Fig. 2a versus Fig. 2e). As the current bias is swept from high negative values through zero, non-equilibrium Joule heating pushes the superconducting transition to a lower current value. This asymmetry decreases as the temperature approaches *T*_c_, where Joule heating effects become less significant (Fig. 2e).

### Effect of an external magnetic field on superconductivity

**Figure 3:**
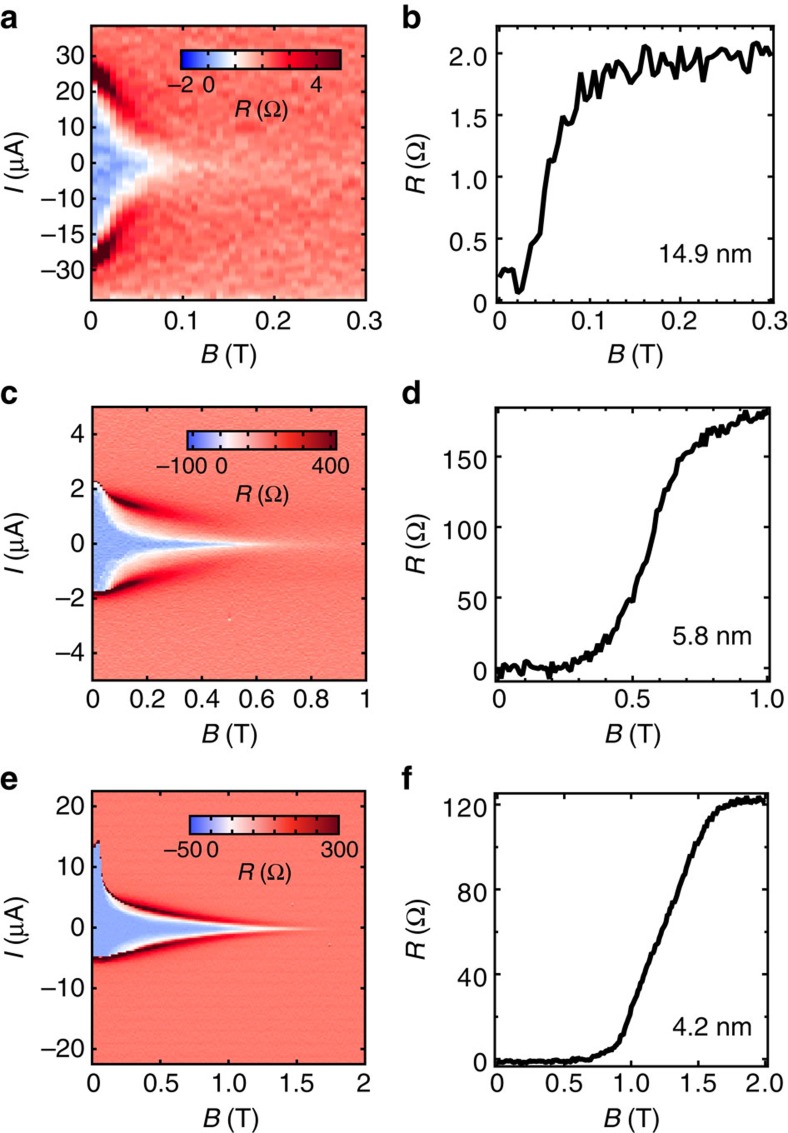
Enhanced critical magnetic field in thin flakes. Perpendicular external magnetic field dependence at 30 mK for three selected devices spanning the range of thicknesses studied. (**a**) Resistance (zero bias numerical derivative) versus applied field for a bulk-like, 14.9 nm device. (**b**) Zero bias resistance versus applied field for the 14.9-nm device. (**c**) Resistance (zero bias numerical derivative) versus applied field for the 5.8-nm device. (**d**) Zero bias resistance versus applied field for the 5.8-nm device. (**e**) Resistance versus applied field for the 4.2-nm device. (**f**) Zero bias resistance versus applied field for the 4.2-nm device.

To further characterize the devices at 50 mK, the upper critical field (*B*_c2_) of these type II superconductors is determined by applying an external magnetic field, perpendicular to the surface of the flake. Figure 3 shows colour scale plots of d*I*/d*V*–*I* curves as a function of external field for the same three devices as in Fig. 2. Figure 3b shows the zero-bias differential resistance as a function of external field. From these curves, we estimate the *B*_c2_ as the external field at which the device returns to the normal-state resistance. Once again, in accordance with the upper critical field reported for the bulk material (110 mT), we measure a *B*_c2_ of ≈130 mT for the bulk-like 14.9 nm flake^49^. The thinner flakes present higher upper critical fields of ≈0.9 T (5.8 nm) and ≈1.7 T (4.2 nm) following the interesting trend for *T*_c_. The critical fields at 50 mK allow estimation of the superconducting Ginzburg–Landau coherence lengths given by: *B*_c2_(50 mK)=*φ*_0_/2*πξ*(50 mK) (ref. 2). The coherence lengths for the 4.2 and 5.8 nm flakes are 13.9 and 19.1 nm, respectively, suggesting that these flakes are in the 2D limit. To further qualify the 2D nature of the thinnest flakes, we analyse the *I*–*V* and *R*–*T* curves (such as those in Fig. 2) of selected devices at zero external field in order to infer the typical signature of 2D superconductivity: the Berezinskii–Kosterlitz–Thouless transition (see Supplementary Fig. 7 and Supplementary Note 3 for details). Note that this study can only be carried out for selected thinner samples for which sufficient data are available. We find that the transport data are consistent with a Berezinskii–Kosterlitz–Thouless superconducting transition, further supporting the 2D nature of the thinnest TaS_2_ flakes.

### Effect of dimensionality on the superconducting state

**Figure 4:**
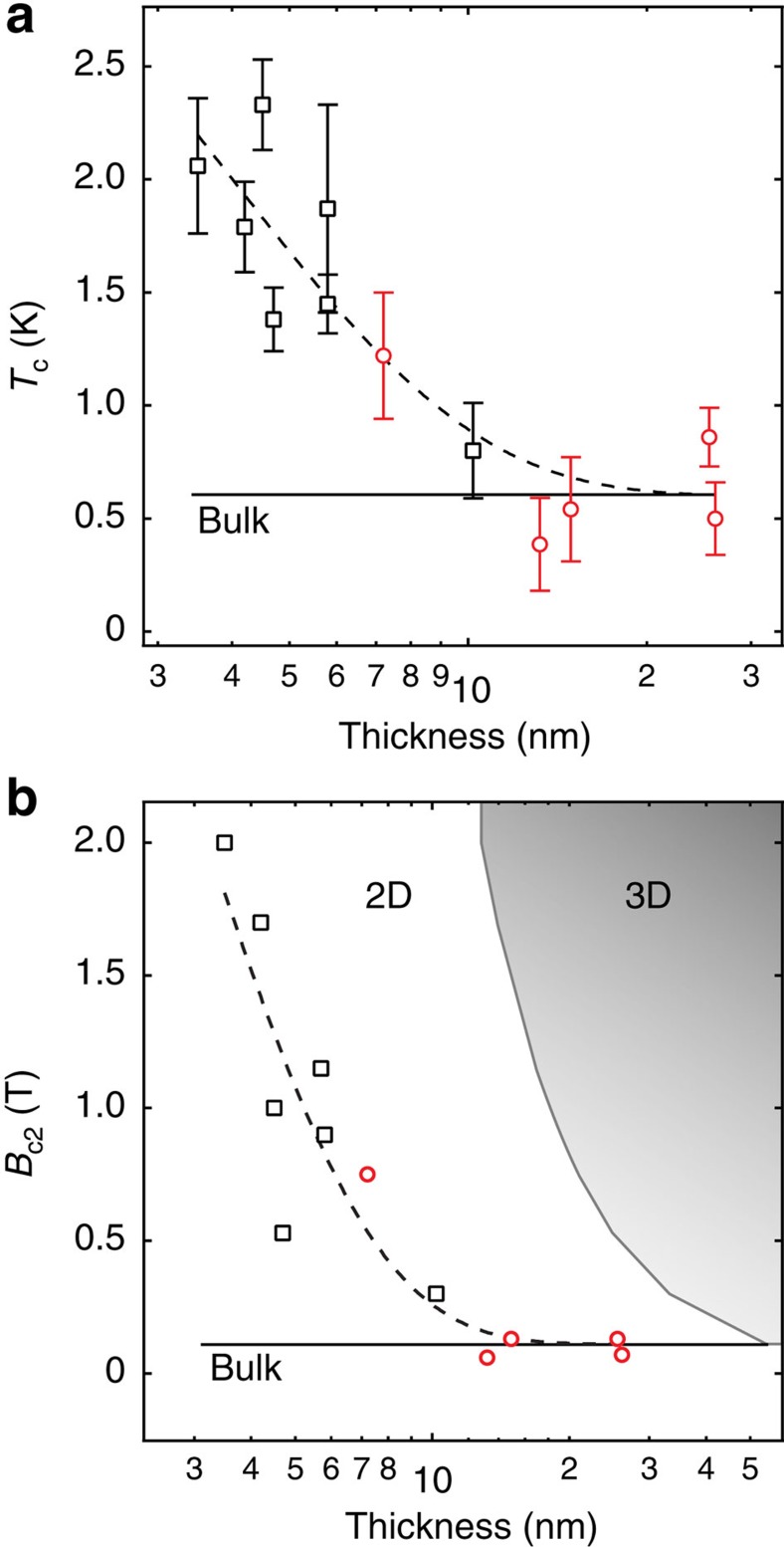
2D superconductivity and enhanced *T*_c_ in atomically thin TaS_2_. (**a**) Variation of *T*_c_ as a function of the thickness of the TaS_2_ flakes. Devices exhibiting a non-zero residual resistance below *T*_c_ are plotted in red. The error bars are given by the temperatures at 10 and 90% of the normal state resistance. The solid black line marks the bulk *T*_c_ of 600 mK. The black dotted line is an exponential trend line, fit to the data starting at the bulk limit. (**b**) Variation of *B*_c2_ as a function of flake thickness. The red circles mark the same devices in **a** having residual resistance. The black solid line indicates the bulk limit upper critical field of 110 mT. The grey solid line plots the GL coherence lengths, calculated from the *y* axis *B*_c2_ values, and marks the edge of the 2D limit.

We now turn our attention to the collective behaviour of our 12 devices and the effect of reduced dimensionality on the superconducting properties of TaS_2_. Figure 4 illustrates the measured *T*_c_ and *B*_c2_ for the devices reported. A bulk limit was found for samples over 10 nm in thickness, such as the one in Figs 2a,b and 3a,b, for which the superconducting properties were consistent with bulk crystals and did not depend on the number of layers. It is interesting to note that these types of flakes exhibit a non-zero residual resistance (red data points) at base temperature, indicating a certain degree of crystalline inhomogeneity and providing a plausible explanation to the slight variation of *T*_c_, similar to the variation in reported bulk values (0.6 and 0.8 K) (refs 25, 50). The bulk-limit devices approach the edge of the 2D limit set by the Ginzburg-Landau (GL) coherence length (*ξ*=55 nm) estimated from the bulk *B*_c2_ (see Fig. 4b).

## Discussion

In addition to thicker flakes that behave in a way consistent with bulk properties, we also observe the superconducting transition in devices made out of thinner TaS_2_ flakes, down to 3.5 nm (∼5 layers). Interestingly, we observe a strong enhancement of *T*_c_ and *B*_c2_ for thinner flakes, up to more than a factor of four larger than in the bulk material. The *T*_c_ enhancement with decreasing number of layers exhibited by the TaS_2_ samples is in strict contrast to the *T*_c_ suppression previously reported in elemental materials^7^, binary systems^51^ and even the closely related dichalcogenide family member, NbSe_2_ (ref. 29). A common theme in these studies is that as the material is thinned down, substrate interactions, either from induced strain or increased Coulomb interactions, suppress the formation of Cooper pairs. In NbSe_2_ devices, a clear correspondence can be made with a decrease in the residual resistance ratio (RRR) giving an indication of increased substrate interactions or more probable that flake degradation is more prevalent in thinner flakes^30^. This agrees with our attempts to contact flakes thinner than 3.5 nm showing a complete insulating state at room temperature. Correspondingly, the RRR values (see Supplementary Fig. 8 for details) for our TaS_2_ sample set show a significant reduction for the two thinnest flakes. However, devices as thin as 4.5 nm still maintain an RRR of 10, indicating pristine thin samples even below our bulk limit of 10 nm.

An initial point that needs to be addressed once trying to interpret the *T*_c_ enhancement is the possibility of electrochemical doping coming from either the original crystals, or through fabrication processes (lithography resists). Although it is well understood that the *T*_c_ of TaS_2_ crystals is particularly sensitive to intrinsic non-stoichiometric doping^52^, we may rule out this effect coming from the original crystals by having measured a bulk *T*_c_ of ∼0.5 K. Now considering the potential doping coming from environmental or intercalation interactions, Raman spectroscopy provides us with strong evidence of the absence of such processes. In contrast with the remarkable peak shifts displayed by intercalated crystals of 2H-MX_2_ (ref. 53), the Raman spectra of exfoliated flakes presented in the supplement (review Supplementary Figs 3–5) show no significant change in crystal structure for flakes of only four layers. Given that the flakes are not undergoing intercalation through exfoliation or fabrication, there could indeed be some doping coming from surface contamination or from the oxide substrate. However, previous studies show that gate-induced or surface-induced electrostatic doping allows for a carrier density modulation of maximum ca 10^12^ cm^−2^ (ref. 54), which is at least three orders of magnitude lower than the estimated single-layer carrier concentration in these metallic TMDCs (ca 10^15^ cm^−2^) (refs 30, 55). In this line, these doping effects have shown to modulate *T*_c_ in NbSe_2_ by 8% at most^30^. Finally, although substrate interactions have led to the interesting *T*_c_ enhancements found in epitaxial grown FeSe on STO, we rule out such effects as the TaS_2_ flakes presented here are weakly coupled to the substrate. This suggests a deeper mechanism as opposed to simple substrate interaction, intercalation or degradation reported in previous studies.

A possible mechanism at work could be the enhancement of the superconducting properties associated with a suppression of the commensurate CDW order, which is in direct competition with superconducting pairing^19^. This is consistent with the interpretation presented of the enhanced *T*_c_ and *B*_c2_ observed in the studies of intercalation of TMDC, where it is argued that the in-plane chemical doping leads to the suppression of the charge density order, and in certain TMDCs under pressure where the same claim is made^49^,^56^,^57^. To explore the effect of the CDW on the DOS at the Fermi level as a function of reduced thickness, we calculate the DOS from an effective one-orbital tight-binding model and simulate the CDW at a mean field level as a periodic potential that locally shifts the onsite energy (see Supplementary Figs 9 and 10 and Supplementary Note 4 for details). We find that the DOS at the Fermi level is not appreciably affected by the CDW for reduced thicknesses. Ultimately, to determine if such a competition with CDWs could be playing a role, one could search for direct evidence of such suppression in STM studies of thin flakes below the 10-nm bulk limit observed here.

An alternative explanation of the enhanced *T*_c_ could be a change of the band structure of the material in atomically thin flakes. To explore this possibility, we perform DFT calculations and construct a simplified tight-binding model to study the electronic band structure and DOS *ν*_N_(0) as a function of the sample thickness. The results of the calculation can be observed in the Supplementary Figs 11 and 12 and Supplementary Notes 5 and 6. The resulting 2D bands contain hole pockets and show saddle points below the Fermi level. These saddle points give rise to van Hove peaks, whose height increases as the number of layers is decreased, and ultimately diverge in the 2D limit. However, the DOS per layer at the Fermi level *ν*_N_(0) decreases as the number of layer is reduced (see Supplementary Fig. 13 for details). For a simplified model with a constant attractive interaction *V*, the coupling constant, that ultimately determines the *T*_c_, takes the usual BCS value *λ*=*Vν*_N_(0). This behaviour of the DOS would suggest at first an analogous trend of *T*_c_, which does not suffice to explain the experiments. The value of the superconducting gap and *T*_c_ can be influenced by the interactions properties of the material. The effective coupling constant^58^ determining *T*_c_ is given by *λ*_eff_=*λ*–μ*, where *λ* is the electron–phonon coupling constant, and μ*, known as Anderson–Morel pseudo-potential, is a term that represents the renormalized repulsive Coulomb interaction. In usual 3D superconductors characterized by a featureless—hence constant—DOS, the projection on the Fermi level of the high-energy degrees of freedom gives rise to a pseudo-potential of the form μ*=*μ*/(1+*μ* ln(*W*/*ω*_0_)), with *ω*_0_ the characteristic phonon frequency, *W* the system bandwidth and *μ* the bare Coulomb repulsion. In a 2D system, with a DOS characterized by a van Hove singularity near the Fermi level, the renormalization of the bare *μ* can be significantly larger than in a 3D material. This effect is therefore strongly dependent on the number of layers. For a generic DOS *ν*_N_(*ε*), the pseudo-potential takes the form


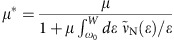


with 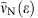 the total DOS normalized with its value at the Fermi energy. Assuming a constant repulsive interaction *U*, one can estimate *μ*=*Uν*_N_(0). For a weak repulsion, the renormalization is negligible and the effective coupling constant follows the DOS at the Fermi level *ν*_N_(0). For a relatively strong Coulomb repulsion, the value of the pseudo-potential at the Fermi level can be strongly affected by features of the DOS at higher energies, such as van Hove singularities. As the number of layers is decreased, the renormalization of a relatively strong repulsion for the band structure in the model is sufficient to reverse the dependence of *T*_c_ on the number of layers obtained from a simple electron–phonon attractive interaction (see Supplementary Fig. 14 for details). This analysis points to a non-negligible role of the Coulomb repulsive interaction in superconducting 2H-TaS_2_, characterized by a predominant Ta 5d orbital character at the Fermi level. The Coulomb repulsion has also been proposed to be at the origin of superconductivity in MoS_2_ (refs 59, 60, 61).

In conclusion, we have reported 2D superconductivity in 2H-TaS_2_ in atomically thin layers. In contrast to other van der Waals superconductors such as NbSe_2_, we find that the *T*_c_ of this material is strongly enhanced from the bulk value as the thickness is decreased. In addition to a possible charge-density wave origin, we propose a model in which this enhancement arises from an enhancement of the effective coupling constant, which determines the *T*_c_. Our results provide evidence of an unusual effect of the reduction of dimensionality on the properties of a superconducting 2D crystal and unveil another aspect of the exotic manifestation of superconductivity in atomically thin transition metal dichalcogenides.

## Methods

### Crystal growth

Polycrystalline 2H-TaS_2_ was synthesized by heating stoichiometric quantities of Ta and S in an evacuated quartz ampoule at 900 °C for 9 days. The growth of large single crystals from the polycrystalline sample was achieved by employing a three-zone furnace. The powder sample was placed in the leftmost zone of the furnace and the other two zones were initially brought to 875 °C and kept at that temperature for 1 day. Following, the temperature of the source zone was risen to 800 °C during the course of 3 h. The temperature of the centre zone was then gradually cooled down at a speed of 1 °C min−1 until a gradient of 125 °C was finally established between the leftmost (875 °C) and centre (750 °C) zones. A gradient of 50 °C was also set between the rightmost and growth zones. This temperature gradient was maintained for 120 h and the furnace was then switched off and left to cool down naturally. The crystals were then thoroughly rinsed with diethyl ether and stored under an N_2_ atmosphere.

### Device fabrication

Contact pads and optical markers are first created on the surface of the Si/SiO_2_ substrates to locate and design contacts to the transferred flakes. The contacts (chromium−5 nm/ gold−70 nm) are then patterned with standard e-beam lithography (Vistec, EBPG5000PLUS HR 100), metal deposition (AJA International) and subsequent lift-off in warm acetone. To preserve the sample integrity, it is crucial to exfoliate, pattern the electrodes and load into the dilution fridge within a few hours. In that respect and even after minimizing the fabrication time, all attempts to contact flakes with thicknesses below 3.5 nm were unsuccessful because of sample degradation.

### Band structure calculations

The DFT simulation of the band structure of 2H-TaS_2_ has been performed using the Siesta code on systems with different number of layers^62^. We use the generalized gradient approximation, in particular, the functional of Perdew, Burke and Ernzerhoff^63^. In addition, we use a split-valence double-*ζ* basis set including polarization functions^64^. The energy cutoff of the real space integration mesh was set to 300 Ry and the Brillouin zone *k* sampling was set, within the Monkhorst–Pack scheme^65^, to 30 × 30 × 1 in the case of multi-layer samples and 30 × 30 × 30 in the case of the bulk calculation. We use the experimental crystal structure of 2H-TaS_2_ for all the calculations^66^.

## Additional information

**How to cite this article:** Navarro-Moratalla, E. *et al*. Enhanced superconductivity in atomically thin TaS_2_. *Nat. Commun.* 7:11043 doi: 10.1038/ncomms11043 (2016).

## Supplementary Material

Supplementary Information Supplementary Figures 1-14, Supplementary Notes 1-6 and Supplementary References
