# Supplementary material for: Enhanced superconductivity in atomically thin TaS2
Source: Nat Commun. 2016 Mar 17;7:11043. doi: 10.1038/ncomms11043 (PMC5512558; doi:10.1038/ncomms11043)
Supplement: Supplementary Information — Supplementary Figures 1-14, Supplementary Notes 1-6 and Supplementary References [file ncomms11043-s1.pdf]

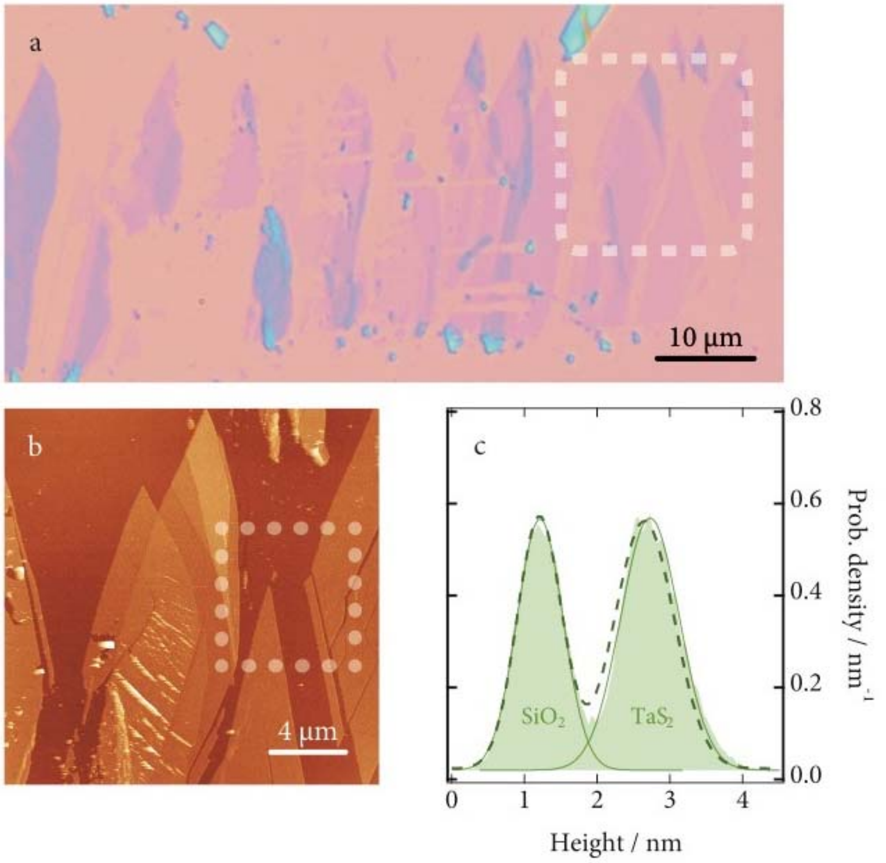

Supplementary Figure 1. Atomically thin TaS_2 flakes deposited on a Si/285 nm SiO2 substrate by the optimised press and shear micromechanical exfoliation method. [a] Optical microscopy image of a region of the substrate displaying a high density of atomically thin flakes. [b] AFM image of the region highlighted in a by the dashed box. [c] Probability density distribution of heights inside the dotted box in b. In this particular image a flake thickness of 1.2 ± 0.5 nm may be estimated.

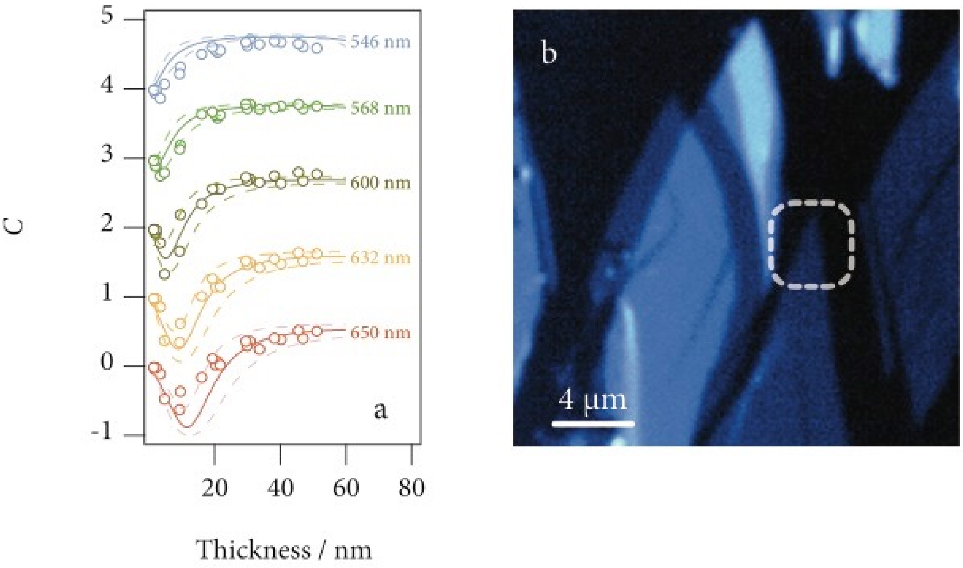

Supplementary Figure 2. Optical contrast study of 2H-TaS$_2$ flakes. [a] Optical contrast (*C*) of a selection of flakes as a function of their thickness under five different monochromatic illumination wavelengths (λ) between 546 nm and 650 nm. The solid lines correspond to the Fresnel-law-model calculation using the refractive index reported in the literature.[7] Note that the sets of contrast measurements for different λ have been shifted vertically by 1, 2, 3 and 4 units for clarity. The uncertainty in *C* due to *a* ± 10% variation in the real and imaginary parts of the refractive index is indicated by the dashed lines. [b] Optical contrast image at λ = 600 nm TaS$_2$ flakes. The flake region marked by the dashed box is 1.2 nm thick, measured by AFM in contact mode, and shows a negative optical contrast of -0.03.

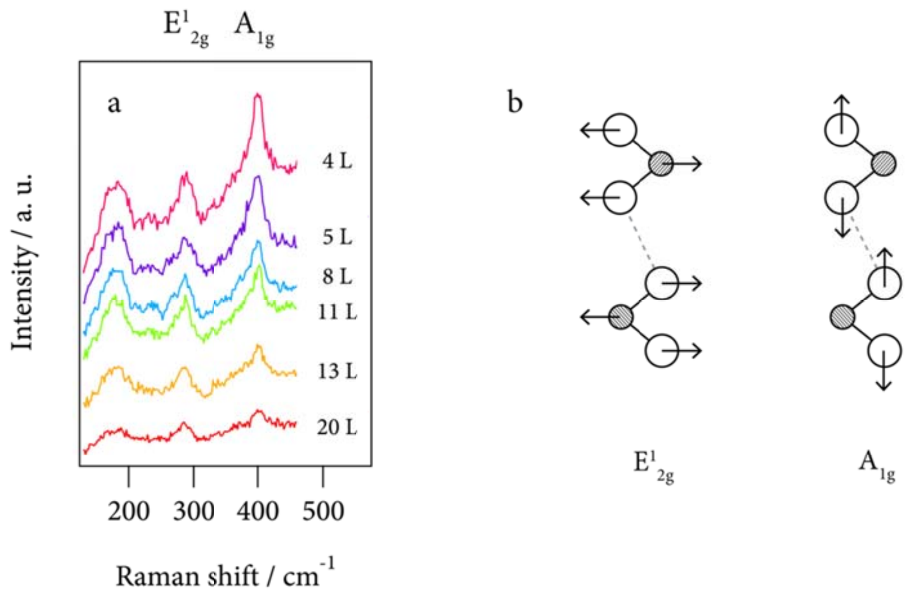

Supplementary Figure 3. Raman spectroscopy of 2H-TaS_2 flakes. [a] Raman spectra measured for 2H-TaS_2 flakes with thickness ranging from four layers to 20 layers. [b] Schematic representation of the vibration modes that correspond to the most prominent peaks at [a].

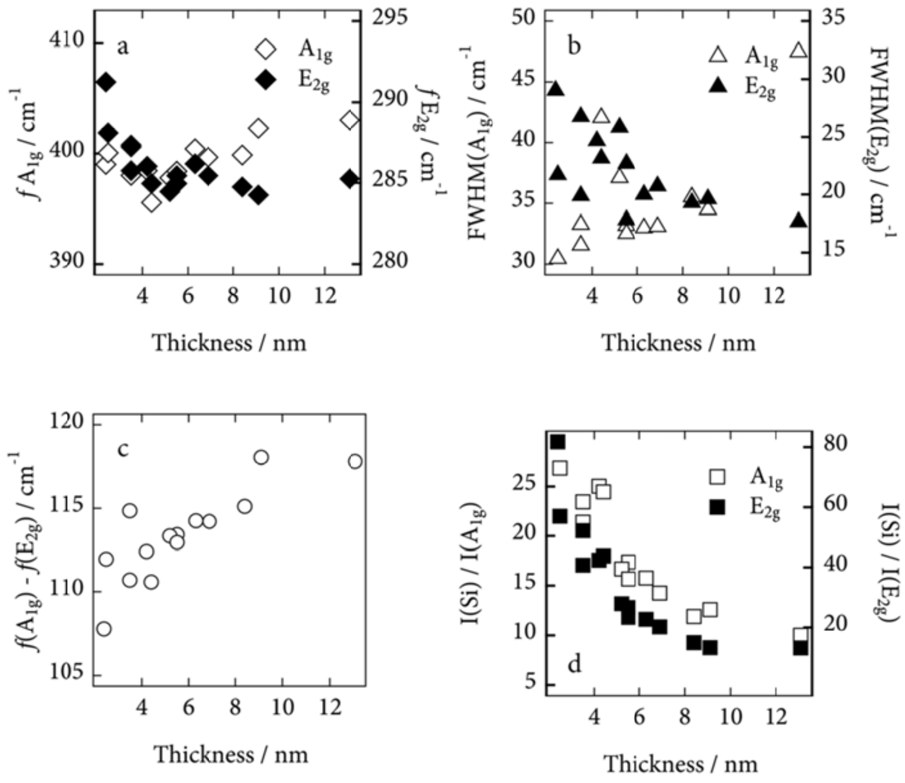

Supplementary Figure 4. Thickness dependence of different Raman features of a selection of 2H-TaS_2 flakes. [a] Frequency shift; [b] FWHM of the A_{1g} and E_{2g} Raman modes; [c] frequency difference between the A_{1g} and E_{2g} Raman modes; and [d] Raman intensity ratio between the Si peak (at 521 cm^{-1}) and the A_{1g} and E_{2g} peaks.

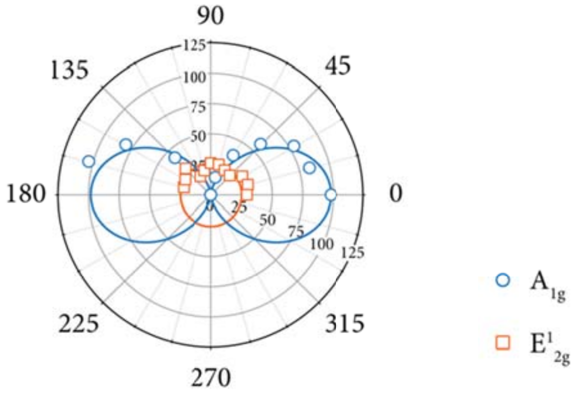

Supplementary Figure 5. Angular dependence of the 2H-TaS_2 signal. Intensity of the A_{1g} and E_{2g} Raman peaks as a function of the angle (in degrees) between linearly polarized excitation and detection.

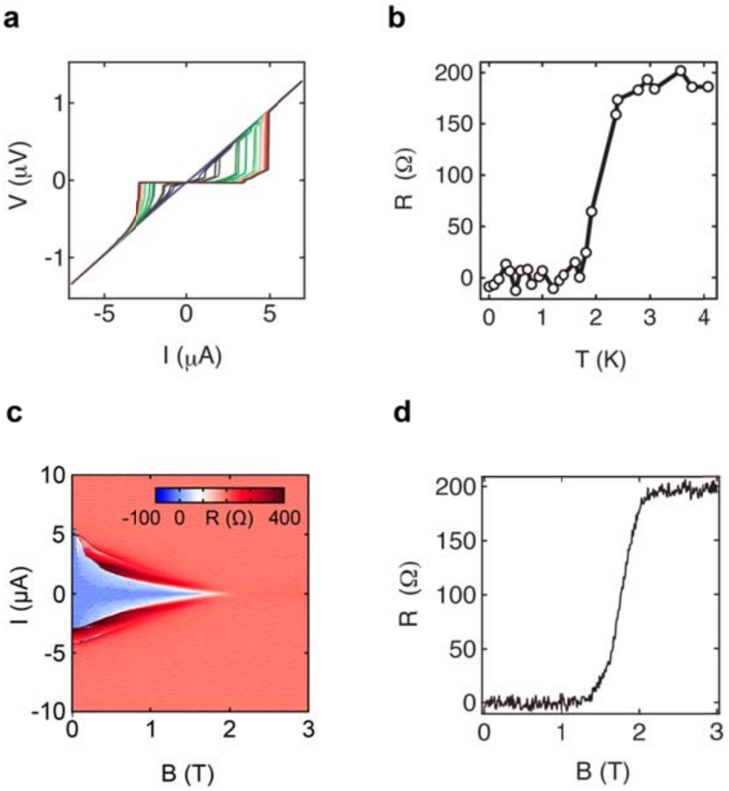

Supplementary Figure 6. Transport properties of a device made out of a 3.5 nm thick TaS_2 flake. [a] Current-voltage (I-V) characteristics as a function of temperature [b] Resistance (zero bias numerical derivative) vs. temperature curve. [c] Resistance vs. applied field and bias current. [d] Zero bias resistance vs. applied field.

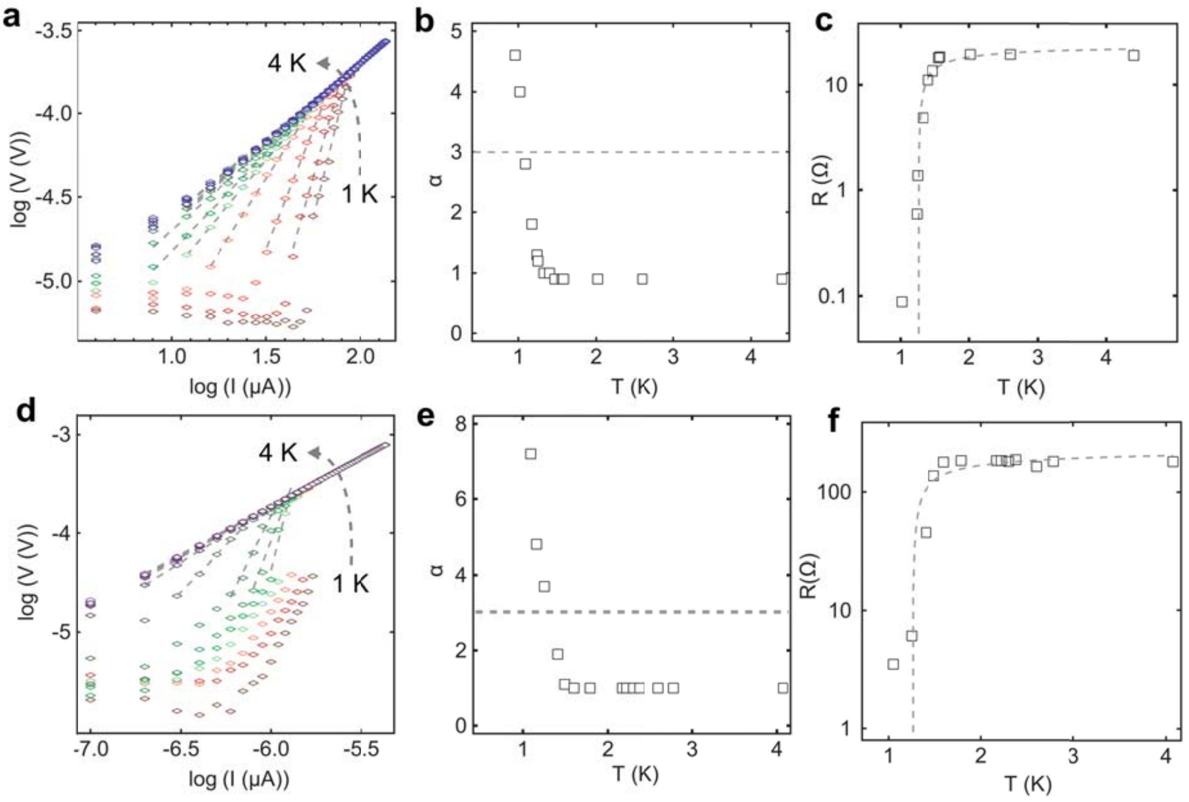

Supplementary Figure 7. Example of a BKT fit performed for a 4.7 nm [a - c] and 5.8 nm [d - e] thick sample. [a, d] *I*-*V* curves are displayed in a log-log scale. [b, e] The variation of the α parameter with temperature as a function of temperature, where the α = 3 value is indicated by the black dashed line and corresponds to T_{BKT}. [c, f] *R*-*T* curves as a function of temperature. The black dotted line plots the *R*(*T*) dependence of the BKT model (see main text).

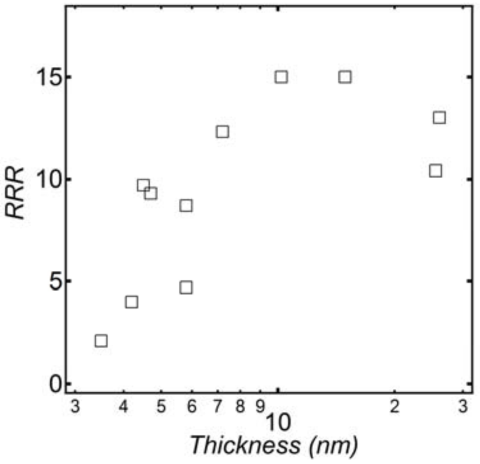

Supplementary Figure 8. Resistance Residual Ratio (*RRR*) as a function of flake thickness. The *RRR* is calculated as the ratio between the room temperature (297 K) resistance and the low temperature resistance at 4 K (*RRR* = *R*(297K)/*R*(4K)). High *RRR* values (~10) are still maintained below the bulk limit thickness of 10 nm indicating pristine flakes and absence of strong substrate interaction.

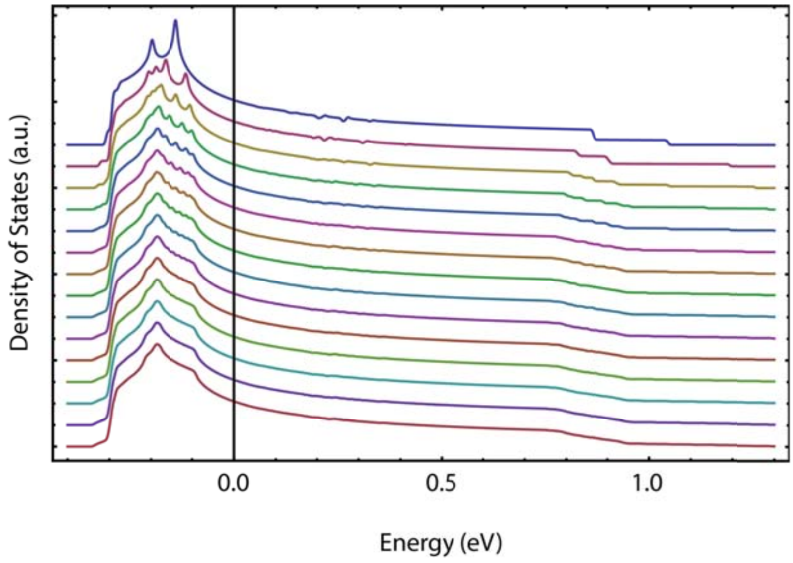

Supplementary Figure 9. Density of states for N=1,…,15 layer 2H-TaS_2 systems in presence of a CDW potential.

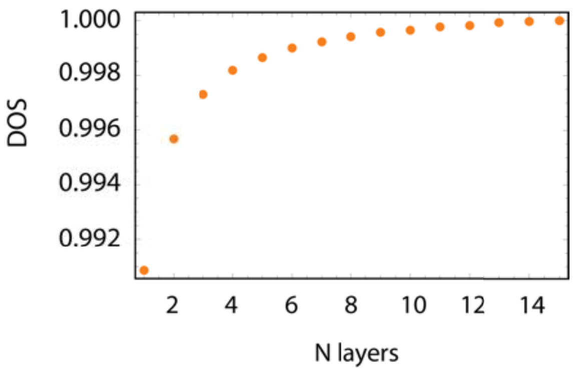

Supplementary Figure 10. Density of states (DOS) at the Fermi level versus N layers of 2H-TaS_2 in presence of the CDW modulation.

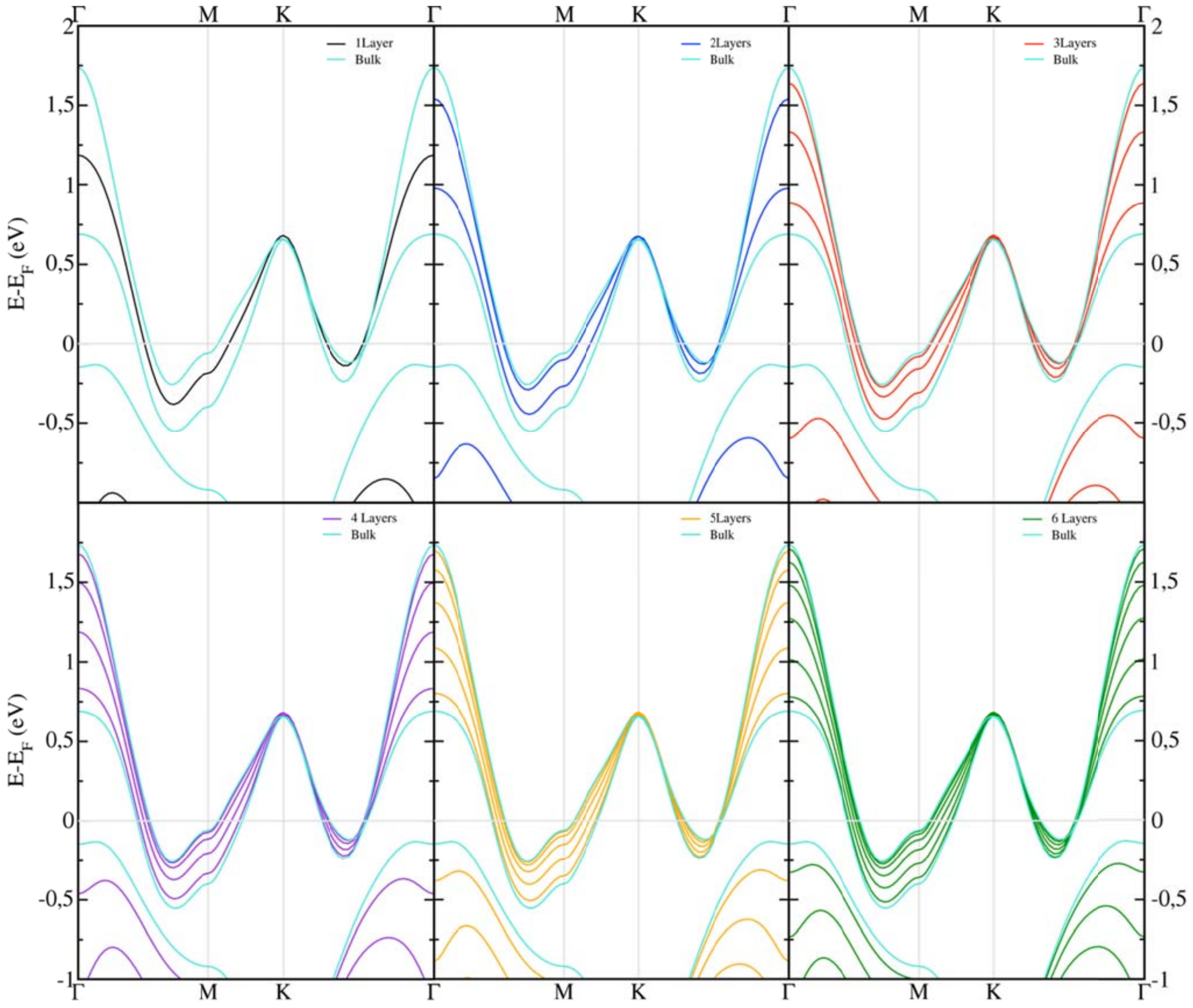

Supplementary Figure 11. DFT band structure of the different systems with varying number of 2H-TaS2 layers from 1 to 6. The bulk band structure (light blue) is plotted in all charts as a reference.

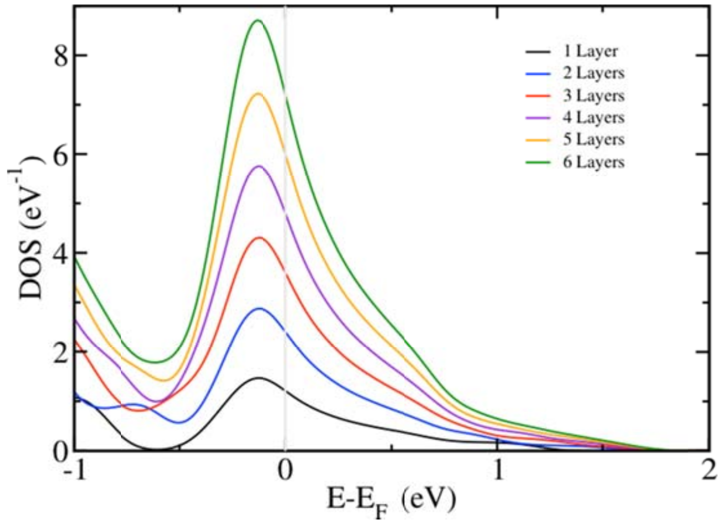

Supplementary Figure 12. Non-normalized DFT calculated density of states (tot-DOS) with varying number of 2H-TaS2 layers.

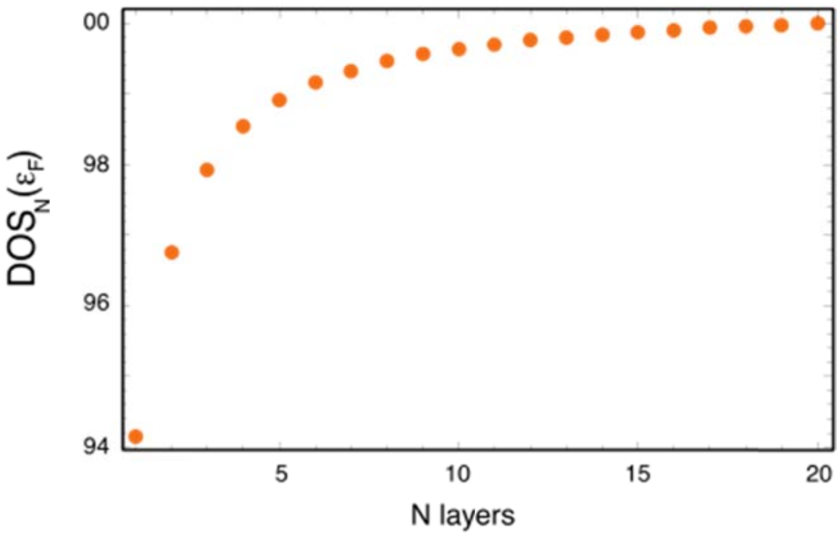

Supplementary Figure 13. Relative change of the Density of States per layer at the Fermi level $\nu_N(0)$ with varying number of TaS_2 layers. The DOS is obtained from the tight-binding model, and the DOS of the N-th layer is normalized to the one with 20 layers.

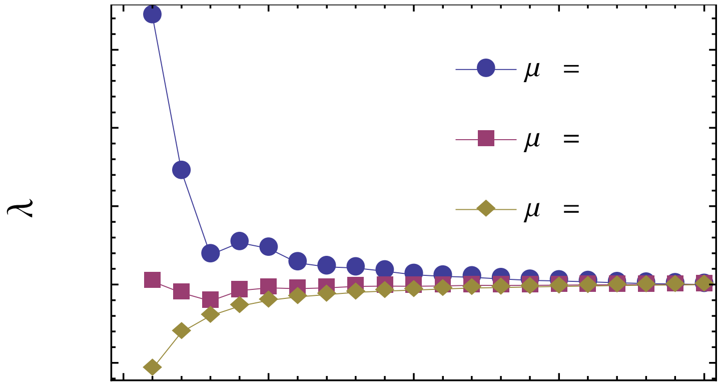

Supplementary Figure 14. Effective coupling constant $\lambda_{\text{eff}} = \lambda - \mu^*$ as a function of the number of layers N, for different values of the bare Coulomb pseudo-potential. In the graph, three values of the bare pseudo-potential are explored ($\mu_{\text{B}} = 0.1, 0.5, 1.5$) and $\lambda_{\text{B}} = 0.36$. λ_{eff} is normalized to its bulk value for a given set of λ_{B} and μ_{B}.

Supplementary Note 1: Optical characterization of 2H-TaS_2 flakes.

The deposition methodology herein described allows for the use of any type of solid substrate as a receiving surface. However, it has already been established how a facile non-destructive detection of atomically thin TMDC layers may be performed by optical microscopy inspection of samples prepared on Si/SiO_2 wafers.[1] Preliminary examination of TaS_2 patches deposited on a Si wafer with a 285 nm thick SiO_2 capping layer permitted the identification of flakes of different thickness flakes as depicted by their different optical contrast (see Supplementary Figure 1).

The presence of this thick silicon dioxide layer between the pure silicon and the deposited material yields an apparent colour that depends on the flake thickness due to a light interference effect.[2] This effect results in very faintly coloured large surface area patches, which correspond to the thinner crystals. In order to quantitatively study the light interference, we measured the thickness dependent optical contrast between the flake and the SiO_2 substrate under different illumination wavelengths (see Supplementary Figure 2).[3456] For accurate determination of the thickness of the deposited TaS_2 flakes, an AFM operated in contact mode was employed. The optical contrast depends both on the flake thickness and the illumination wavelength (shown in Supplementary Figure 2), and can be calculated using a model based on Fresnel laws using the refractive index of TaS_2 reported in the literature.[7] We found a significant agreement between the measured optical contrast for thin flakes and that obtained from the model using the refractive index of bulk TaS_2. It is remarkable that the optical contrast is strongly dependent on the illumination wavelength and even changes its sign for flakes thinner than 20 nm. This behaviour makes white light illumination inappropriate for the identification of the thinnest flakes by optical

microscopy. Oppositely, illumination under certain wavelengths enhances the optical contrast of the thinnest TaS_2 crystals, which allowed the optical identification of layers as thin as 1.2 nm.

Supplementary Note 2: Raman spectroscopy of 2H-TaS_2.

The potential relationship between the flake thickness and the Raman scattering intensity was explored. For this reason a μ-Raman probe was used to explore different thickness flakes. Supplementary Figure 4 shows the thickness dependence of a selection of Raman features. Whereas the frequency shift and the full-width-at-half-maximum (FWHM) of the A_{1g} and E_{2g} Raman modes do not seem to be at all related to the number of layers, it may be clearly appreciated how the ratio between the intensity of the Si peak (at 521 cm^{-1}) and the A_{1g} and E_{2g} peaks both increase upon decreasing the number of layers of the probed flake. The frequency difference between the A_{1g} and E_{2g} Raman modes also exhibit a linear proportionality with the number of layers present in the flakes. It is important to highlight that as for other TMDCs, some sensitivity to the Raman laser beam was also exhibited by the TaS_2 flakes. In this way, upon performing experiments with long exposure times or high irradiation powers, the flakes were irreversibly damaged as seen by a change in the optical contrast in the focus spot of the laser beam. Yet, no apparent change in the height profile as measured by AFM could be detected. By contrast, the appearance of a strong photoluminescence emission band around 555 nm suggested that some oxidation to Ta_2O_5 had occurred.[8]

On a final note, it has been previously observed in other transition metal dichalcogenides how the intensity of the distinct Raman modes may vary as the angle between the linearly polarized incident beam and the scattered signal is modified.[9] This can be used to confirm the origin of the Raman peaks. In the TaS_2 case, it could be observed that while the intensity of the E_{2g} mode does not depend on the angle between the excitation and detection, the A_{1g} mode presents its maximum intensity for parallel excitation and detection and it vanishes for cross polarized

excitation and detection in agreement with that reported for other TMDC flakes,[10] confirming that the Raman signal comes from an analogous crystal (see Supplementary Figure 5).

Supplementary Note 3. Berezinskii–Kosterlitz–Thouless (BKT) fits to selected devices.

I-V curves were fit to a power law of the form $V \propto I^{\alpha}$, where α spans from 1 for temperatures above T_{BKT}, reaching a value of $\alpha = 3$ at the BKT transition, and monotonically increasing as temperature is further lowered. The R-T curves are further fit to the resistance dependence near the BKT temperature, $R = R_N \exp(-b/(T-T_{BKT})^{1/2})$. Supplementary Figure 7 shows a set of *I-V* and *R-T* curves taken for two devices with thicknesses of 4.7 nm [a – c] and 5.8 nm [d - f]. It may be appreciated how the data do follow a power law with values of the α parameter that vary in the expected range typical for 2D superconductivity. From the α-exponent analysis and *R-T* transition we estimate a BKT temperature of 1 K for the 4.7 nm flake and 1.2 K for the 5.8 nm flake.

Supplementary Note 4. Charge density wave (CDW) considerations in 2H-TaS_2.

The experimentally observed charge density wave in 2H-TaS_2 has a periodicity of 3 x 3 unit cell in the layer plane. We consider an effective one-orbital tight-binding model and simulate the CDW at mean field level as an onsite potential that locally shift the onsite energy.

The effect of the CDW is seen in the DOS at an energy of 0.2 - 0.3 (in units of the tight binding hopping parameters), in the form of small corrugation arising from the gap opening in part of the band structure (Supplementary Figure 9).

At the Fermi energy, no gap is opened (again visible in Supplementary Figure 9) and the behavior of the DOS at the Fermi level (Supplementary Figure 10), which is ultimately responsible of the T_c, is not affected by the presence of the CDW.

Supplementary Note 5. DFT band structure and tight-binding model.

A crucial starting point for studying the behavior of the critical temperature versus the thickness of the sample is a faithful description of the system in terms of a band structure and wavefunctions. The details of the calculations are described in the Methods section of the main text.

The resulting band structure of our calculations is shown in Supplementary Figure 11. The bands crossing at the Fermi level have a strong Ta d character. The Fermi surface for a system composed by N layer is constituted by N pockets around Γ, N pockets in K, and N pockets in K', and in the limit of large N give rise to the well known tubular Fermi surfaces. The calculated total density of states (tot-DOS) is shown in Supplementary Figure 12, where we can see that it presents a large peak slightly below the Fermi level, whose height increase linearly with increasing number of layers, and whose position does not change when varying the number of layers. The tot-DOS at the Fermi level also presents a linear increase with increasing number of layers. From the band structure we understand the peak as arising from van Hove singularities associated to saddle points in the band structure at the M point and at an intermediate point between the K point and Γ point. A van Hove peaks is a logarithmic singularity that shows up in the DOS when the system is 2D. As we increase the number of layers the van Hove singularities get smooth and average out when the systems becomes 3D.

The meshing in the DFT simulations, although sufficiently dense for the convergence of the total energy, is not sufficient to resolve the van Hove peak in the DOS. We then construct an effective tight-binding model of a single orbital in a triangular lattice considering in-plane and out-of-plane hopping up to second nearest neighbors,

$$H_0 = -\sum_{n,m=1}^{N} \sum\nolimits_{i,j} c_{i,n}^{*}\, t_j^m c_{i+j,n+m} + H.c.$$

and by fitting the DFT band structure we find $t_1^0 = -0.033$, $t_2^0 = -0.227$, $t_0^1 = -0.039$, $t_1^1 = -0.016$, $t_2^1 = -0.010$, where t_j^n is the hopping matrix element between the n-th out-of-plane and the j-th in-plane nearest neighbor sites. The 2nd in-plane nearest neighbor hopping t_2^0 comes out to be larger than the 1st nearest neighbor one, a result that has been already discussed in the literature,[11] and is due to a phase cancelation mechanism, typical of 2H-TMDCs. The resulting DOS per layer at the Fermi level $\nu_N(0)$ is shown in Supplementary Figure 13, where we clearly see a monotonic decrease of the DOS as we lower the number of layers.

Supplementary Note 6. Anderson-Morel model.

The Anderson-Morel model takes into account the effect of a repulsive Coulomb interaction that reduces the effective coupling constant determining the superconducting T_c. Here, we generalize the Anderson-Morel model to a system with a generic DOS, that allows us to properly account for the van Hove logarithmic singularities appearing as the systems becomes more and more 2D, as is the case when lowering the thickness of the sample. The idea is to correctly project to the low energy sector the contribution of the high energy repulsive tail of the effective electron-electron interaction. The starting point is a generalized gap equation in the framework of the Eliashberg theory that couples the gap at all energies,

$$\Delta(\varepsilon) = -\int d\varepsilon' V_{\mathrm{eff}}(\varepsilon,\varepsilon')\nu_N(\varepsilon')\frac{\tanh\left(\frac{\varepsilon'}{2T}\right)}{2\varepsilon'}\Delta(\varepsilon')$$

where the effective interaction $V_{\mathrm{eff}}(\varepsilon,\varepsilon')$ is the result of electron-phonon and the electron-electron terms. To keep the problem as simple as possible, we follow the usual treatments and discretize the effective interaction as $\nu_N(0)V_{\mathrm{eff}}(\varepsilon,\varepsilon') = -\lambda + \mu$, for $-\omega_0 \leq \varepsilon, \varepsilon' \leq \omega_0$, and $\nu_N(0)V_{\mathrm{eff}}(\varepsilon,\varepsilon') = \mu$, for $-W \leq \varepsilon, \varepsilon' \leq W$, with W the bandwidth of the DOS. This way, the bandwidth interval is characterized by two regions with different interactions, attractive at a low energy and repulsive at high energy. The gap function can then be separated in two values in the different two regions, $\Delta(\varepsilon) = \Delta$ for $|\varepsilon| < \omega_0$, and $\Delta(\varepsilon) = \Delta\xi$, for $\omega_0 < |\varepsilon| < W$. By introducing the bulk quantities $\lambda_{\mathrm{B}} = V\,\nu_{\mathrm{bulk}}(0)$, $\mu_{\mathrm{B}} = U\,\nu_{\mathrm{bulk}}(0)$, the gap equation simplifies to the following system of two coupled equations for Δ and ξ,

$$1 = (\lambda_{\mathrm{B}} - \mu_{\mathrm{B}})I_1 - \mu_{\mathrm{B}}\,\xi\,I_2$$
$$\xi = -\mu_{\mathrm{B}}\,I_1 - \mu_{\mathrm{B}}\,\xi I_2$$

where we have defined the following integrals,

$$I_1 = \int_0^{\omega_0} d\varepsilon \frac{\tanh(\varepsilon/2T)}{\varepsilon} \tilde{\nu}_N(\varepsilon) \qquad\qquad I_2 = \int_{\omega_0}^{W} d\varepsilon \frac{\tanh(\varepsilon/2T)}{\varepsilon} \tilde{\nu}_N(\varepsilon)$$

with $\tilde{\nu}_{\rm N}(\varepsilon) = (\nu_{\rm N}(\varepsilon) + \nu_{\rm N}(-\varepsilon))/2\nu_{\rm bulk}(0)$. As we have seen by the DFT simulations and the tight-binding model, the total DOS normalized by the number of layer is featureless close to the Fermi level, so that the integral I_1 is performed in the usual way and it gives $I_1 = \ln(1.14\ \omega_0/T)$. At the same time, the DOS displays van Hove singularities at higher energies, that become more and more pronounced as we lower the number of layers. In the limit $T/\omega_0 \ll 1$, the integral I_2 can be approximated as

$$I_2 = \int_{\omega_0}^{W} d\varepsilon \frac{\tilde{\nu}_N(\varepsilon)}{\varepsilon}$$

Using the approximate DOS resulting from the tight-binding model, we checked that the value of I_2 monotonically increases with lowering the number of layers, even if the van Hove peaks are smoothed in the numeric approximations. The effective coupling constant $\lambda_{\rm eff} = \lambda - \mu^*$ is then written as,

$$\lambda_{\rm eff} = \nu_N(0) \left(\lambda_{\rm B} - \frac{\mu_{\rm B}}{1 + \mu_{\rm B}\, I_2} \right)$$

where the dependence on the number of layers N is hidden in $\nu_N(0)$ and I_2. As we pointed out in the main text, the renormalization of the pseudo-potential is particularly relevant if the bare Coulomb term is sufficiently strong. In Supplementary Figure 14 we plot the effective coupling constant $\lambda_{\rm eff}$ for three different values of the bare pseudo-potential, $\mu_{\rm B} = 0.1, 0.5, 1.5$. Since we are interested in the trend of $\lambda_{\rm eff}$ with lowering N, we choose $\lambda_{\rm B}$ greater than the bulk value of

$1/I_2$, so to guarantee a positive coupling constant. In the computation of I_2 we choose the phonon frequency $\omega_0 = 50$ meV and for $\lambda_{\mathrm{B}} = 0.36$. For weak value of μ_{B}, the effective coupling constant follows the DOS at the Fermi energy, whereas for stronger repulsion we can clearly see that λ_{eff} increases.

SUPPLEMENTARY REFERENCES

[1] a) Li, H., Lu, G., Yin, Z., He, Q., Li, H., Zhang, Q. & Zhang, H. Optical Identification of Single- and Few-Layer MoS2 Sheets. *Small* **8**, 682–686 (2012). (b) Castellanos-Gomez, A., Navarro-Moratalla, E., Mokry, G., Quereda, J., Pinilla-Cienfuegos, E., Agraït, N., van der Zant, H.S.J., Coronado, E., Steele, G.A. & Rubio-Bollinger, G. Fast and reliable identification of atomically thin layers of TaSe2 crystals. *Nano Research* **6**, 1-9 (2013).

[2] Kvavle, J., Bell, C., Henrie, J., Schultz, S. & Hawkins, A. Improvement to reflective dielectric film color pictures. *Optics Express* **12**, 5789 (2004).

[3] Blake, P., Hill, E. W., Castro-Neto, A. H., Novoselov, K. S., Jiang, D., Yang, R., Booth, T. J. & Geim, A. K. Making graphene visible. *Applied Physics Letters*, **91**, 063124 (2007).

[4] Castellanos-Gomez, A., Agrait, N. & Rubio-Bollinger, G. Optical identification of atomically thin dichalcogenide crystals. *Applied Physics Letters*, **96**, 213116 (2010).

[5] Castellanos-Gomez, A., Wojtaszek, M., Tombros, N., Agrait, N., van Wees, B. J. & Rubio-Bollinger, G. Atomically Thin Mica Flakes and Their Application as Ultrathin Insulating Substrates for Graphene. *Small*, **7**, 2491-2497 (2011).

[6] Benameur, M.M., Radisavlievic, B., Héron, J. S., Sahoo, S., Berger, H. & Kis. A. Visibility of dichalcogenide nanolayers. *Nanotechnology* **22**, 125706 (2011).

[7] Beal, A.R., Hughes, H. P. & Liang, W. Y. The reflectivity spectra of some group VA transition metal dichalcogenides. *Journal of Physics C: Solid State Physics*, **8**, 4236-4234 (1975).

[8] Zhu, M., Zhang, Z. & Miao, W. Intense photoluminescence from amorphous tantalum oxide films. *Appl. Phys. Lett.* **89**, 021915 (2009).

[9] Wu, Y., An, M., Xiong, R., Shi, J. & Zhang, Q. M. Raman scattering spectra in the normal phase of 2H–NbSe2. *J. Phys. D: Appl. Phys.* **41**, 175408 (2008).

[10] a) Plechinger, G., Heydrich, S., Eroms, J., Weiss, D., Schuller, C. & Korn, T. Raman spectroscopy of the interlayer shear mode in few-layer MoS2 flakes. *Appl. Phys. Lett* **101**, 101906 (2012). (b) Plechinger, G., Heydrich, S., Hirmer, M., Schrettenbrunner, F. X., Weiss, D., Eroms, J., Schuller, C. & Korn, T. Scanning Raman spectroscopy of few- and single-layer MoS2 flakes. *Proc. SPIE, Nanoengineering: Fabrication, Properties, Optics, and Devices IX* **8463**, 84630N (2012).

[11] Guillamón, I., Suderow, H., Rodrigo, J. G., Vieira, S., Rodière, P., Cario, L., Navarro-Moratalla, E., Martí-Gastaldo, C. & Coronado, E. Chiral charge order in the superconductor 2H-TaS2. *New J. Phys*. **13**, 103020 (2011)
